# Establishing the size and configuration of the imaging support workforce: a census of national workforce data in England

**DOI:** 10.1093/bjro/tzae026

**Published:** 2024-09-05

**Authors:** Julie Nightingale, Sarah Etty, Beverley Snaith, Trudy Sevens, Rob Appleyard, Shona Kelly

**Affiliations:** Centre for Applied Health and Care Research (CARe), Sheffield Hallam University, Sheffield, S10 2BP, United Kingdom; Centre for Applied Health and Care Research (CARe), Sheffield Hallam University, Sheffield, S10 2BP, United Kingdom; Faculty of Health Studies, University of Bradford, Bradford, BD7 1DP, United Kingdom; Department of Radiology, Mid Yorkshire Teaching NHS Trust, Wakefield, WF1 4DG, United Kingdom; Centre for Applied Health and Care Research (CARe), Sheffield Hallam University, Sheffield, S10 2BP, United Kingdom; Centre for Applied Health and Care Research (CARe), Sheffield Hallam University, Sheffield, S10 2BP, United Kingdom; Centre for Applied Health and Care Research (CARe), Sheffield Hallam University, Sheffield, S10 2BP, United Kingdom

**Keywords:** support worker, assistant practitioner, workforce, imaging, radiology, radiography

## Abstract

**Objectives:**

The imaging support workforce is a key enabler in unlocking imaging capacity and capability, yet no evidence exists of the workforce size and configuration. This research provides the first comprehensive analysis of workforce data to explore the deployment of the support workforce within National Health Service (NHS) imaging services in England.

**Methods:**

Using a census methodology, an anonymized electronic staff record (ESR) data set extracted in December 2022 was analysed to identify support workers and their employment bandings at NHS Trust, regional and national (England) level. Support workforce proportions, median values, and Spearman’s rank correlations were calculated.

**Results:**

Analysis of 137 NHS Trusts, comprising 100% of acute trusts (*n* = 124) and specialist trusts with imaging services (*n* = 13), identified that the support workforce (pay bands 2-4) constitutes 23.6% of the imaging staff base. Ranking trusts into 3 categories based on the proportion of support workers in their imaging establishment, median values ranged from 30.7% (high) to 22.2% (medium) and 10.5% (low). Two opposing deployment models of band 2 and band 3 support workers were identified.

**Conclusions:**

Comprising almost one-quarter of the imaging establishment, models of deployment at bands 2 and 3 are highly variable. Assistant practitioners (band 4) are under-utilised, providing an opportunity to introduce innovations to address workforce demands.

**Advances in knowledge:**

This census is the first to provide evidence of the size and structure of the support workforce, the first step in enabling effective workforce transformation. Further research is required to explain the two opposing deployment models.

## Introduction

Demand for diagnostic imaging services in England is rising year on year, against a backdrop of persistently high vacancy rates in the registered workforce (radiographers and radiologists).^1^^,^^2^ In 2023 alone, an unsustainable £276 million was spent by National Health Service (NHS) imaging services on insourcing (overtime), outsourcing (to private companies), locum and agency staff to fill in the service gaps.^1^ There is an urgent need for expansion of the imaging workforce, using efficient and effective skills mix strategies to ensure that their scope of practice is maximized. While radiographer skills mix (including enhanced, advanced, and consultant practice) is now embraced, the imaging support workforce has historically not been a focus for development.^3^^,^^4^ However 3 pivotal national reports published in 2019/2020 (Diagnostics: Recovery and Renewal^4^; Transforming Imaging Services in England: a national strategy for imaging networks^5^; Radiology GIRFT Programme National Specialty Report^6^) signalled an urgent need to develop the capacity and capability of the imaging support workforce to support effective skills mix.

In 2021, the Allied Health Professions Support Worker Competency, Education and Career Development Framework was launched to reduce unwarranted variation and ensure these roles are “…at the heart of improvements in service delivery and transformation, including new models of care”.^7^ The revised College of Radiographer’s Education and Career Framework for the Radiography Workforce (2022) highlights that with appropriate supervision the support workforce can undertake many patient-facing activities, including image acquisition, that were formerly in the domain of the registered radiographer.^8^ This releases time for registrants to undertake vital advanced and enhanced roles such as image reporting, providing additional capacity to support service delivery and enable radiologists to undertake and report on the most complex imaging procedures.

The UK imaging support workforce is now modelled on a four-tiered structure of clinical support workers, senior clinical support workers, associate practitioners, and assistant practitioners (APs) (Table 1).^8–10^ These tiers correspond to bands 2-4 in Agenda for Change (AfC), the system used by the NHS to structure staff pay,^11^^,^^12^ and collectively will be referred to in this article as support workers and assistant practitioners (SWAP). APs are well-established within breast screening,^13^ with increasing numbers of mammography associate roles also emerging, yet within other areas of imaging, centres are yet to employ APs or continue to restrict their deployment to specific imaging modalities.

**Table 1.** UK imaging support workforce structure.

Tier	Clinical support worker (CSW)	Senior clinical support worker	Associate practitioner (mammography)	Assistant practitioner (AP)
Level	Entry level	Intermediate	Advanced	Advanced
Typical qualifications/apprenticeship levels	FHEQ^a^ level 2 (secondary school level, eg, GCSEs), Care Certificate	FHEQ level 3 (college level, eg, A Levels); profession-appropriate qualification	FHEQ level 4. (higher education level, eg, Cert HE^b^ or Mammo Assoc. Apprenticeship)	FHEQ level 5 (higher education level, equivalent to a Dip HE^c^ or Foundation Degree)
Typical grade (agenda for change^d^)	Pay band 2	Pay band 3	Pay band 4	Pay band 4/5
Supervision	Close supervision. Report directly to a registered practitioner	Direct or indirect supervision when required	Under the supervision of a registered Radiographer	Work semi-autonomously within a specified care plan, under supervision of registered staff. Supervision model varies depending on area of work, experience, and scope of practice
Role	Enables effective patient care. Important clerical, administrative, housekeeping tasks to support delivery of imaging services.	As CSW, but also clinical support and care before, during and after imaging examinations. Range of delegated duties, including clinical tasks.	Care for women in breast screening programme or with breast cancer symptoms, operate specialist mammography equipment.	Competently performs noncomplex examinations in areas previously within the remit of a registered professional, working to locally agreed standard operating procedures, protocols, or systems of work. Work is protocol-driven within defined scope of practice.
Example imaging tasks	Supporting patients to change clothing; assist infection control process; managing stock and supplies; reception duties	Intravenous cannulation; patient positioning; support preparation of contrast agents and procedure trolleys	Perform routine 2-view mammography in hospital/mobile breast screening unit, supports quality assurance	Primarily patient-facing. General radiography or mammography exams within imaging department setting or breast screening service. Support patients during invasive procedures/complex pathways or provide aseptic scrub support

a Agenda For Change: https://www.healthcareers.nhs.uk/working-health/working-nhs/nhs-pay-and-benefits/agenda-change-pay-rates.

b FHEQ—Framework for Higher Education Qualifications: https://www.qaa.ac.uk/the-quality-code/qualifications-frameworks.

c Cert HE—Certificate of Higher Education.

d DipHE—Diploma of Higher Education.

**Figure 1.**
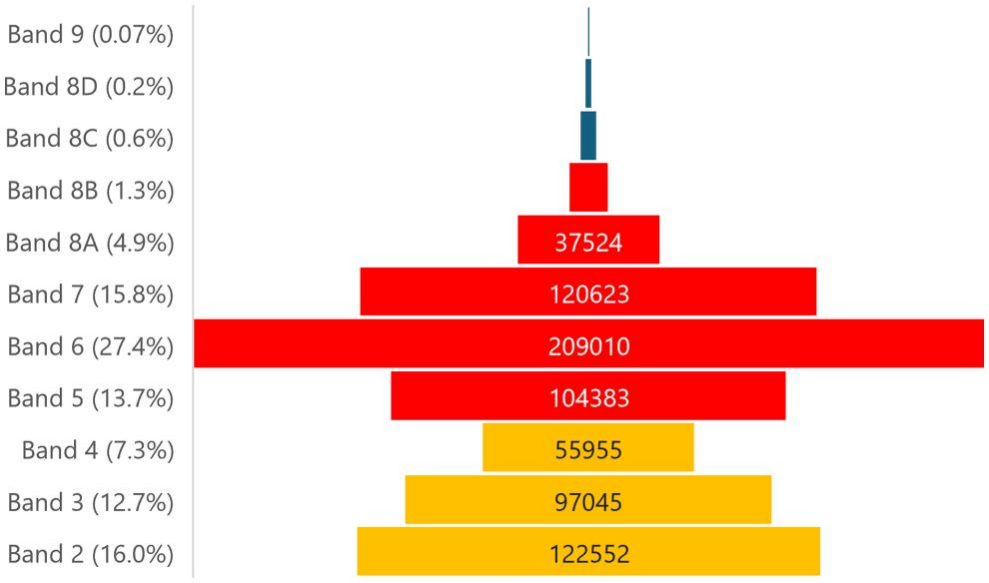
Distribution of the NHS Clinical Workforce (excluding doctors) across pay bands (whole time equivalents). Adapted from Imeson et al^10^ and NHS England.^12^ The support workforce (bands 2-4) comprises 36.1% of the nonmedical workforce.

An analysis of the distribution of the whole NHS clinical workforce shows that clinical support workers make up approximately 36.1% of the nonmedical staffing, with both the registered workforce (band 5 and above) and the unregistered SWAP workforce (bands 2-4) distributed in a double pyramid staffing structure (Figure 1).^12^^,^^14^^,^^15^ This model presents a wide-based platform on which to strategically develop the SWAP workforce to take on new roles, enabling the registered staff to extend their skills from within their own wide base. However, it is unknown whether imaging departments mirror this staffing structure, and therefore whether there is sufficient capacity and capability within the imaging support workforce to support current policy ambitions.^4^^,^^16^ Currently available data relating to the imaging workforce, for example, through NHS England’s Model Health System,^17^ is incomplete and is unavailable at the individual level of analysis; however, initial review highlights substantial variability in the deployment of SWAPs. This variability is not explained by research evidence; no articles have been published relating to imaging SWAP workforce structures in England,^3^^,^^18^ and a very weak evidence base exists for the impact and effectiveness of AP roles.^18–24^ Most other studies are over a decade old,^20–23^^,^^25–28^ and deployment practices are likely to have progressed. If the support workforce is to be effectively mobilized to underpin imaging transformation, a comprehensive understanding of the size and configuration of the current establishment is required. The aim of this research was to undertake a census of national imaging workforce data to explore the deployment of the support workforce within imaging services in England.

## Methods

This article reports on the first phase of an explanatory mixed methods research programme which aims to investigate the current deployment, development, and contribution of the NHS imaging SWAP workforce within England. Each subsequent phase in the ‘I-SWAP’ (Imaging SWAP) study will contribute to the creation of a determinant framework^29^ centred upon effective models of skills mix and the factors which are likely to influence their implementation. The relative size and structure of the imaging SWAP workforce were determined by analysis of an anonymized Health Education England (HEE) workforce data set drawn from the Electronic Staff Record (ESR). The ESR, commissioned by the Department of Health and Social Care, is a payroll database system used by 99% of NHS Trusts to manage the payroll for over 1.8 million NHS employees.^30^ As data extraction took place at a single point in time (December 2022), this is a snapshot of the actual workforce in employment on that date. Nevertheless, this provides us with the first national census of the size and configuration of the support workforce. Institutional ethics approval was gained Sheffield Hallam University (ID:ER46621650) and following completion of a data sharing agreement, gatekeeper permission from HEE was also granted. The project complied with best practice in NHS research ethics and governance via compliance with the UK Policy Framework for Health and Social Care Research (2023).^31^

### Data extraction and cleansing

A census methodology (complete enumeration) was used to collect and analyse the data, which included both whole time equivalents (WTE) and person counts for the entire NHS imaging workforce in England, excluding nursing and medical staff. WTE is a standardized measure of the workload of an employed person and allows for the total workforce workload to be expressed in an equivalent number of full-time staff; 1.0 WTE equates to full-time work of 37.5 hours per week. The anonymized data provided on each person included the region of the country, organization, job role, occupation code, and area of work; no personal data such as names or identification numbers were supplied. As these data are regarded as a complete count of the imaging workforce, it is not relevant to calculate a sample size.

At the time of data extraction, there were 229 NHS Trusts in England, including acute, specialist, community, mental health, and ambulance trusts. The ESR data set included all trusts with imaging services (*n* = 143); however, during initial data extraction, 6 NHS Trusts were immediately excluded as their ESR records indicated the provision of endoscopy services only or services with no radiographers or support workers. The remaining data set (*n* = 137 NHS Trusts) represented all (100%) of the 124 NHS acute trusts in England, in addition to some specialist trusts with imaging services. Initial analysis showed a high level of variation in data presentation (such as job titles); therefore, piloting was undertaken of a single HEE region to establish comprehensive and reproducible inclusion and exclusion criteria for the wider dataset (Table 2). The ESR records of both diagnostic radiographers and imaging-focused support workers were included, whereas medical staff, medical physicists, healthcare scientists, nurses (including bands 2-4 healthcare assistant and nurse associate roles), and managers were excluded. To reduce high levels of variability, staff records linked to Nuclear Medicine were excluded as in some centres this modality did not fall within the traditional ‘Radiology’ umbrella. Similarly, sonographers who were employed by midwifery and/or healthcare science departments, rather than radiology, were excluded.

**Table 2.** Inclusion and exclusion criteria (imaging department electronic staff records).

Excluded staff records	Included staff records
Medical staff (radiologists)	Radiographers—diagnostic (bands 5-8)
Medical physicists	Radiographer—diagnostic, specialist practitioner
Healthcare scientists	Radiographer—diagnostic advanced practitioner
Nurses	Consultant radiographer
Radiology service managers and operational managers	Sonographers (bands 5-8)
Sonographers indicated as ‘midwife’ or ‘healthcare science practitioner’	Instructor/teacher/clinical tutor/educator
Therapeutic radiographers/therapists	Imaging or radiology support worker/assistant (bands 2-3)
Nuclear medicine staff	Assistant practitioner (band 4+)
Students/trainees	Student/trainee radiographers (if band 4+)—assumed to be awaiting HCPC registration
Health care assistants (HCAs) within general nursing, adult nursing, and non-clinical support staff (porters, reception staff)	Health care assistants (HCAs) within imaging modalities (CT/ultrasound/X-ray/MRI, etc)

### Data analysis

Following exclusion of ineligible staff, the remaining staff WTEs were allocated to individual pay bandings and to one of 2 groups, the SWAP workforce (bands 2-4) or the radiographer workforce (bands 5-8). For each NHS Trust, the proportion of support workers within the entire imaging workforce (SWAPs + radiographers) for that location was calculated, with the Trusts ranked in order of proportion of SWAPs. This ranking was stratified into approximately equal thirds (high/medium/low SWAP proportions). As data were non-normally distributed, the median and interquartile range were calculated for each of these categories. Additional correlations explored the relationships between the different work groups and pay bands. This included a correlation of the proportions of band 4 posts with advanced and consultant posts (bands 7 and 8), providing an indication of whether SWAPs might be an enabler of advanced practice.

## Results

### Total imaging and support workforce across England

**Figure 2.**
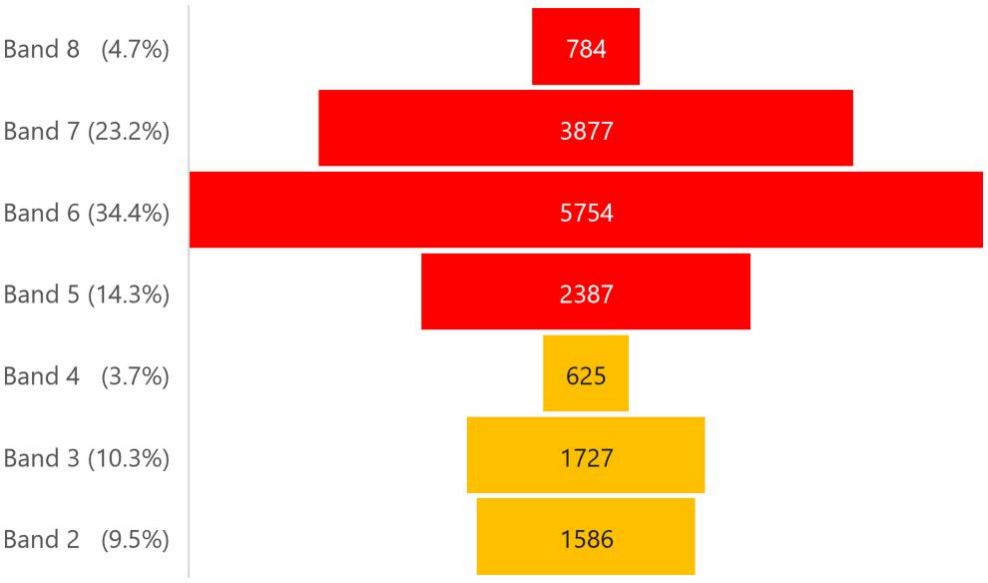
Distribution of the imaging workforce (whole time equivalents) in England across NHS pay bands (*n* = 137 NHS Trusts, data extracted from Electronic Staff Records December 2022). The support workforce is represented in bands 2-4.

The analysed data set included ESR data from 137 NHS trusts. A combined total of 12 842.5 WTE radiographers (bands 5-8) and 3961.9 WTE support workforce (bands 2-4) were identified; based on these WTE values, SWAPs comprised 23.6% of the entire imaging workforce (median 22.2%, IQR 14.9-29.1). The distributions of the imaging workforce across the different AfC pay bands^9^ are seen in Figure 2.

When trusts were ranked into 3 categories based on their SWAP proportions, the median values ranged from 30.7% (high) to 22.2% (medium) and 10.5% (low). The proportion of advanced and consultant radiographers (bands 7 and 8) within the total radiographer workforce was also calculated (median 36.6, IQR 28.9-47.5) (Table 3).

**Table 3.** Proportions for each role (whole time equivalents) for NHS Trusts placed within 3 categories (high, medium, low support workforce proportions).

Calculation	All trusts combined	High	Medium	Low
Support worker proportion (%)	22.17 (14.87-29.12)	30.72 (29.14 -33.45)	22.17 (20.68-23.97)	10.51 (1.98-14.81)
Total imaging workforce size (WTE)	97.88 (57.27-176.66)	120.56 (68.77-192.72)	111.26 (71.55-177.59)	65.38 (36.73-132.54)
Advanced and consultant proportion (%)	36.56 (28.93-47.51)	32.09 (26.81-42.83)	36.56 (29.47-43.18)	42.41 (31.78-61.56)

Figures presented as median (IQR).

### Analysis of grade distributions

**Figure 3.**
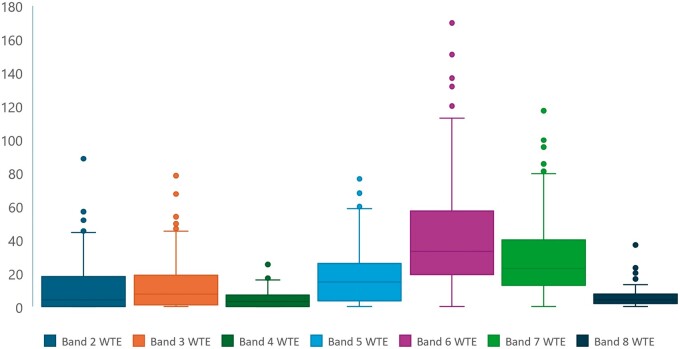
Box and whisker plot to illustrate the median values and IQRs of whole time equivalent (WTE) staff (*y*-axis) within each pay band for a typical imaging department (*n* = 137 NHS Trusts, data extracted from Electronic Staff Records December 2022).

Data from all NHS Trusts were combined to produce the median values for each pay banding. A box and whisker plot (Figure 3) illustrates the median and IQR values of the workforce structure of an ‘average’ imaging establishment.

**Figure 4.**
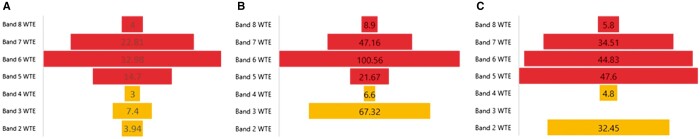
Workforce configurations across pay bandings based on whole time equivalent (WTE) numbers within each band. (A) Median combined values (*n* = 137 NHS Trusts) to demonstrate a ‘typical’ imaging department workforce configuration. The support workforce is represented in bands 2-4. (B) WTE counts for a selected NHS Trust imaging department which does not utilize band 2 support workers (model A). (C) A selected NHS Trust with no deployment of band 3 support workers (model B). Data extracted from Electronic Staff Records December 2022.

Review at the individual NHS Trust level demonstrates that there is significant variation in the grades that SWAPs occupy. Figure 4 depicts the different configurations by workforce shape and WTE numbers of staff occupying each grade. Figure 4A depicts a representation of the ‘typical’ imaging workforce size and structure based on combined NHS Trust data. However, review at the individual organizational level reveals that some imaging departments employ either high proportions of band 2 (model A) or band 3 support workers (model B), rather than a combination of the two grades. Figure 4B and C illustrates the extremes of these contrasting models within two selected NHS Trusts. Model A (Figure 4B) is an imaging department with ∼250 imaging staff, deploying no band 2 support workers and very few band 4 APs and band 5 registered radiographers. Model B (Figure 4C) is an imaging department with ∼170 imaging staff, deploying no band 3 support workers, few band 4 APs, but a wide band 5 deployment. Other NHS Trusts lie between these two extremes, though usually favouring deployment of either band 2 or 3 support workers. APs (band 4) appear to be employed in small numbers by some but not all imaging departments, equating to 15.9% of the total SWAP workforce.

### Support workforce proportions by imaging department size and region

**Figure 5.**
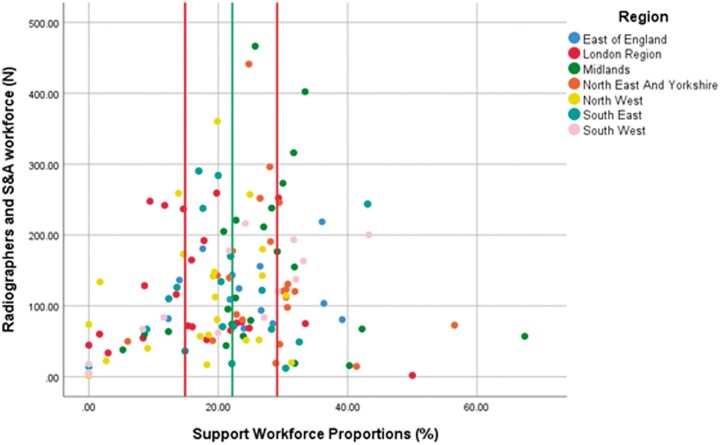
Scatter plot charting the support workforce proportion (*x*-axis) against imaging workforce size (*y*-axis) for each NHS Trust in England. Notes: Individual trusts (dots) are colour coded for the region in which they reside. The median value is displayed (green line) and the IQR indicated (red lines).

Figure 5 displays the SWAP proportion for each of the NHS Trusts plotted against the entire imaging workforce (radiographers and SWAPs combined). Each Trust is colour coded to represent the region of England in which the NHS Trust resides. This very wide scatter plot illustrates wide variability in the size of the support workforce as a proportion of the imaging establishment; this does not appear to be directly influenced by the size of the imaging department, though larger imaging departments are more clustered around the median value than the smaller departments which tend to have a smaller SWAP proportion. There are no trends identified across the different regions, though the South East region has the least variability.

### Support workforce correlation with other staff groups

**Figure 6.**
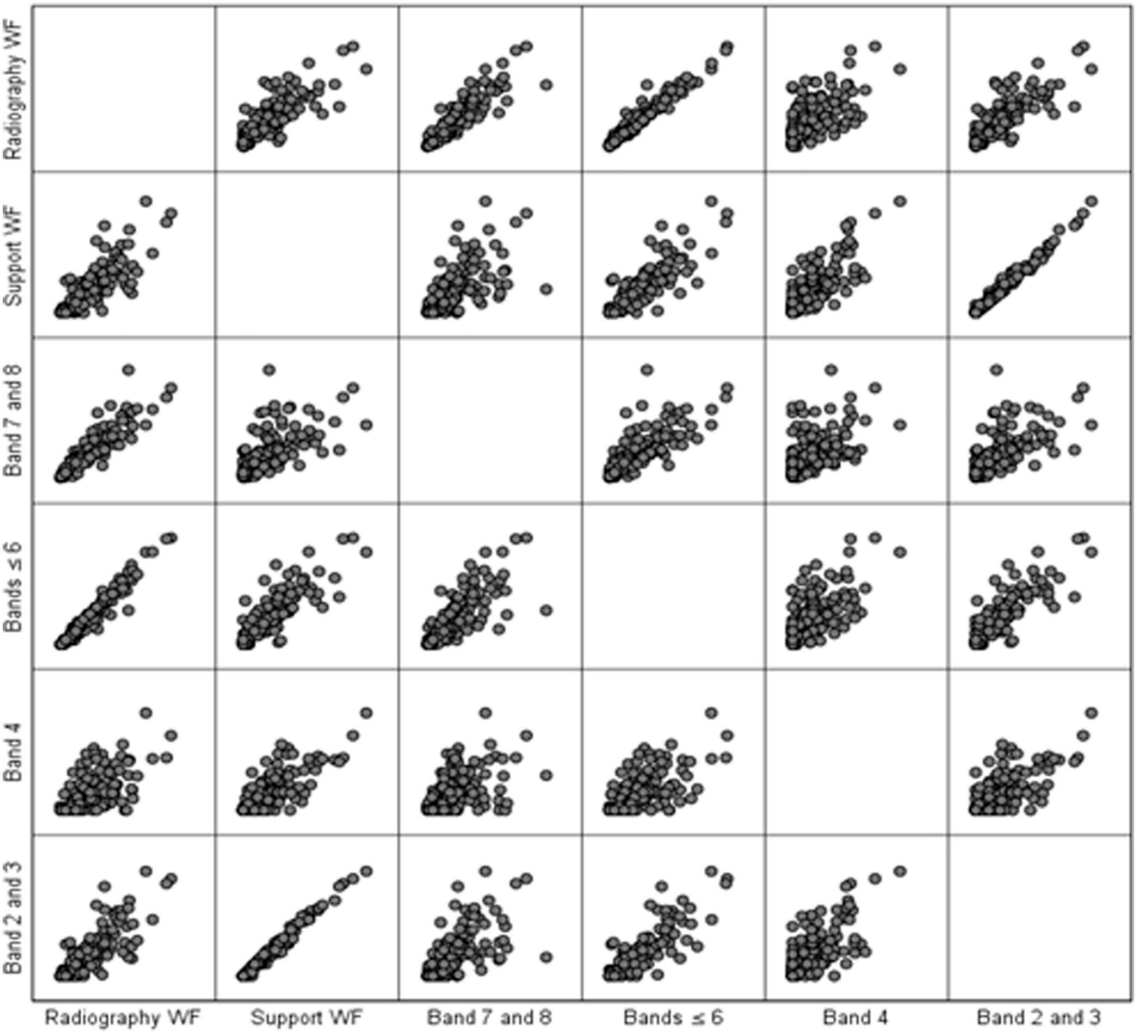
Relationships between different staff grades (scatter plots).

Spearman’s rank correlations of the different staff groups were calculated to the data set, as the data were not normally distributed (Table 4, Figure 6). These relationships show a statistically significant (.01 level, 2-tailed) and strong (>.5) positive correlation, although caution should be applied as relationships between the groups of variables within variables would be expected. The correlation between the number of band 4 support workers and band 7 and 8 radiographers was at .546 the weakest correlation within this table.

**Table 4.** Relationships between different staff grades (Spearman’s rank correlations).

Staff group	1	2	3	4	5	6
1. All radiographers (bands 5-8)	–					
2. Support workforce (bands 2-4)	.812*	–				
3. Band 7 and 8 radiographers	.912*	.701*	–			
4. Band 5 and 6 radiographers	.963*	.820*	.784*	–		
5. Band 4 assistant practitioners	.638*	.760*	.546*	.644*	–	
6. Band 2 and 3 support workers	.794*	.986*	.687*	.801*	.659*	–

* Significant at <.001 level. Correlations of band 7/8 radiographers (indicated to be advanced and consultant practitioners) are shown in bold.

## Discussion

The imaging support workforce is a vital component in underpinning wider imaging workforce transformation.^4–6^ Having the right number of staff with the appropriate skills and qualifications is a critical determinant of the quality and efficiency of health care,^12^ yet there is currently a very limited understanding of the capability and capacity of the imaging support workforce. This census provides the first comprehensive compilation of national imaging workforce data to explore the deployment of the support workforce within diagnostic imaging services in England.

Analysis of ESRs from NHS Trusts in England (*n* = 137) identified a combined total of 12 842.5 WTE radiographers (bands 5-8) and 3961.9 WTE support workers (bands 2-4). Health and Care Professions Council (HCPC) registrant figures from the same period show a much higher total of 43 040 radiographers^32^; however, this is individual registrants, whereas the WTE number will include part-time staff. Additionally, the HCPC figure also includes both diagnostic and therapeutic disciplines from across the United Kingdom, includes radiographers working outside the NHS, for example, in academia and the independent health sector, and overseas registrants not working within the United Kingdom. The radiographer numbers are considered to be an accurate reflection of the NHS radiography workforce in England. Our analysis identified that SWAPs (bands 2-4) comprise approximately one-fifth of the imaging workforce (Median 22.27%, IQR 14.9-29.1). The most recent Society of Radiographers Diagnostic Radiography Workforce UK Census Report highlighted a slightly lower mean support workforce proportion of 21.1%,^2^ although this figure was based on responses from only 47 NHS Trusts. Support workers comprise a much smaller proportion of the imaging workforce when compared to the wider NHS clinical workforce (36.1%, Figure 1),^12^^,^^14^^,^^33^ suggesting that there may be scope to expand within imaging.

The support workforce proportion varies widely between imaging departments; when ranked into different SWAP ‘adopter’ categories, their median proportions ranged from 30.7% (high) to 22.2% (medium) and 10.5% (low). Smaller imaging departments appeared to have greater variability in SWAP proportions than larger centres which appeared to be more clustered around the mean value. This suggests that for a larger establishment to be effective there is a ‘rule of thumb’ or guiding principle for a balance between support staff and radiographers. The opportunity for flexibility is not available to smaller departments who tend to have lower SWAP proportions. Regional differences were not apparent, although the South-East region has the lowest variability in SWAP proportions.

An insight into the capacity of the imaging workforce can be seen by modelling the workforce against their Agenda for Change pay bandings.^11^ The visual representation of the imaging workforce shape and structure (Figure 2) does not align comfortably with the ‘double pyramid’ configuration seen in the wider NHS clinical workforce (Figure 1).^12^^,^^14^ When data from all NHS Trusts are combined, the imaging support workforce appears to lack the wide base from which to underpin workforce transformation^12^; bands 2 and 3 have similar proportions, with a much smaller than expected band 4 category. Surprisingly, analysis at the individual trust level shows that few imaging departments have similar proportions of band 2 and 3 support workers, electing instead to deploy support workers predominantly at either band 2 or band 3. Failure to employ band 2 support workers may lead to challenges in recruiting applicants with sufficient clinical skills and experience for band 3 roles, while failure to employ band 3 staff may restrict career progression of band 2 support workers with consequent retention issues, reducing the opportunities for skills mix in modalities where higher-level skills such as cannulation is required.

Review of the imaging workforce structure (Figure 2) demonstrates that the registered workforce also lacks the broad base seen in the wider NHS registered workforce^12^^,^^14^; the band 5 category is much smaller in comparison to band 6. Linked gradings that support accelerated progression from band 5-6 within 2 years (Agenda for Change Annex 20)^34^ may account for this apparent disparity. While the imaging workforce structure in Figure 2 is a visual representation of the entire imaging workforce in England, it provides a model against which individual imaging departments may compare their support and registered imaging workforce, providing an insight into the current establishment and the potential for workforce planning and development.

AP are normally deployed at band 4, comprising 3.7% of the entire imaging workforce (*n* = 625), and 15.9% of the clinical support workforce (Figure 2). This is a much lower utilization of band 4 APs than is seen in the wider NHS (7.3% and 20.3%, respectively),^12^^,^^14^ suggesting that this may be a focus for future imaging workforce development. However, the evidence base for the impact and cost effectiveness of imaging APs beyond breast screening is weak,^3^ which may be limiting wider adoption across other modalities. APs have a wider scope of practice than other support workers, and this includes the acquisition of images in non-complex settings that may release radiographers for enhanced and advanced practice.^7–10^ Any correlation between the proportion of band 4 APs and band 7 and 8 radiographers is therefore potentially of interest. While there is a statistically significant (.01 level, 2-tailed) and strong (>.5) positive correlation [41] at .54, this is a weaker correlation than those of other staff groups. Given the small numbers of APs deployed within the majority of imaging services, it is unlikely that the presence of APs directly enables advanced practice, despite the aspirations of the original radiography skills mix strategies,^35^^,^^36^ though this may occur in some settings such as breast screening.

This census has highlighted wide variations in deployment, with many imaging departments either not fully embracing, or under-utilizing, the support workforce. This is a missed opportunity, as the imaging support workforce is large and highly flexible, and central to delivery of imaging transformation and the wider NHS Long Term Workforce Plan.^4–6^^,^^16^ Imeson et al^12^ outline several advantages of developing the support workforce to promote wider workforce transformation, with short training times meaning that numbers can be expanded rapidly, and their good quality, patient-focused care reducing the workload of highly qualified staff.^12^ Development of support roles can deliver more rewarding roles and enhanced career pathways, improving retention, and through apprenticeships they widen participation for those who do not have academic qualifications to become professionally qualified.^12^ Ultimately, the support workforce can deliver greater efficiencies resulting in benefits to both patients and NHS organizations in addressing workforce capability and capacity and reducing reliance on expensive agency staff.

The analysis highlighted several important limitations. The ESR data records only staff in the post rather than the funded establishment for each imaging department so does not indicate vacancy levels or locum appointments. The ESR data were found to be incomplete, with a wide range of job titles which made coding difficult. The original purpose of the ESR was to indicate the employee’s salary for payroll and record their qualifications for the role,^30^ but evolution of job titles over time has occurred without a requirement to update the records of existing staff in those roles. Job titles below band 4 were highly variable and the research team had to work with several data fields to place a person into the correct category. In extracting the data for analysis, workforce analysts in HEE and the research team therefore had to make several documented judgement calls which assisted in standardizing decision-making for the wider data set (see Table 2). Data extraction took place in December 2022, and therefore should be considered a ‘snapshot in time’ which may not accurately reflect the current support workforce. While recognizing these limitations, our analysis nevertheless appears to reflect the more limited, self-reported workforce statistics provided within the latest Society of Radiographers workforce census report.^2^

## Conclusion

This research represents the first national census of the imaging support workforce in England, presenting a comprehensive compilation and analysis of payroll data (*n* = 137 NHS Trusts) identified through the review of the NHS ESR. Deployment of imaging staff does not follow the staffing structure seen in the wider clinical NHS workforce, with a narrower support worker base than is required to underpin effective skills mix and service transformation. Support workforce deployment is highly variable and does not appear to be influenced by overall workforce size or region, though larger departments may have greater opportunity and flexibility in support worker deployment. While data at a national level demonstrate an imaging support workforce with similar proportions of band 2 and 3 support workers, individual analysis of imaging departments highlights examples of two distinct models of deployment of either band 2 or band 3 support workers, reducing the potential for the support workforce to underpin wider workforce transformation. There is clearly scope within most departments to increase the deployment of APs.

Radiology service managers are urged to review the size and configuration of their support workforce, comparing their workforce structure to the combined and individual models presented in this analysis. This will provide evidence to support their future workforce planning and remodelling initiatives. The implications of under-utilization and highly variable deployment highlighted in this census should be urgently reviewed by workforce leaders and policy-makers at organization, system, national (commissioners and professional bodies), and international levels, informing policy initiatives to ensure imaging workforce transformation delivers greater efficiencies and maximizes patient care and staff experience. However, this research raises important questions related to how and why support workers are utilized across different pay bands, modalities, and imaging settings (eg, specialist, acute, community), and what barriers and enablers exist for effective deployment. The ‘I-SWAP’ research team subsequently embarked upon a follow-on qualitative study to explore these research questions and identify examples of innovations that can support future workforce remodelling and transformation.
